# Medicaid Subscription-Based Payment Models and Implications for Access to Hepatitis C Medications

**DOI:** 10.1001/jamahealthforum.2021.2291

**Published:** 2021-08-27

**Authors:** Samantha G. Auty, Paul R. Shafer, Kevin N. Griffith

**Affiliations:** Department of Health Law, Policy, and Management, Boston University School of Public Health, Boston, Massachusetts; Department of Health Law, Policy, and Management, Boston University School of Public Health, Boston, Massachusetts; Department of Health Policy, Vanderbilt University School of Medicine, Nashville, Tennessee

## Abstract

**IMPORTANCE:**

Hepatitis C virus (HCV) can be cured with direct-acting antiviral medications, but state Medicaid programs often restrict access to these lifesaving medications owing to their high costs. Subscription-based payment models (SBPMs), wherein states contract with a single manufacturer to supply prescriptions at a reduced price, may offer a solution that increases access. Whether SBPMs are associated with changes in HCV medication use is unknown.

**OBJECTIVE:**

To estimate changes in Medicaid-covered HCV prescription fills after Louisiana and Washington implemented SBPMs on July 1, 2019.

**DESIGN, SETTING, AND PARTICIPANTS:**

This cross-sectional study examined trends in prescription fills of Medicaid-covered direct-acting antiviral HCV medications in Louisiana and Washington after implementation of SBPMs. A synthetic control approach was used to compare changes in HCV prescription fills between states that did and did not implement SBPMs. The unit of analysis was state-quarter. Outpatient direct-acting antiviral HCV prescription fills from the Medicaid State Drug Utilization Data files were obtained from all 50 US states and the District of Columbia from January 1, 2017, to June 30, 2020.

**EXPOSURES:**

Implementation of SBPMs for Medicaid-covered direct-acting antiviral HCV medications.

**MAIN OUTCOMES AND MEASURES:**

Direct-acting antiviral HCV prescriptions filled per 100 000 Medicaid enrollees.

**RESULTS:**

In the year preceding SBPM implementation, the mean (SD) rate of quarterly HCV prescription fills per 100 000 Medicaid enrollees was 43.1 (8.6) prescriptions in Louisiana and 50.1 (4.1) in Washington. After SBPM implementation, the mean (SD) rate of quarterly HCV prescription fills per 100 000 enrollees was 206.0 (51.2) prescriptions in Louisiana and 53.9 (11.0) in Washington. In synthetic control models, SBPM implementation in Louisiana was associated with an increase of 173.5 (95% CI, 74.3-265.3) quarterly prescription fills per 100 000 Medicaid enrollees during the following year, a relative increase of 534.5%(95% CI, 228.7%-1125.0%). Washington did not experience a significant change in prescription fills following SBPM implementation.

**CONCLUSIONS AND RELEVANCE:**

In this cross-sectional study, Louisiana experienced substantial increases in HCV medication use among its Medicaid-enrolled population following SBPM implementation, whereas Washington did not. These differences may partially be explained by state-level variation in SBPM implementation, historical restrictions on access to HCV medications, and responses to the COVID-19 pandemic.

## Introduction

Hepatitis C virus (HCV) affects an estimated 2.4 million people in the United States, and the rate of new infections has risen more than 250% in the last decade.^1^ In response, the Centers for Disease Control and Prevention has endorsed universal screening for HCV in all adults.^2^ In tandem, guidelines published by the Infectious Diseases Society of America recommend treatment for all patients with acute or chronic HCV, but timely access to treatment remains elusive for many.^2,3^ Treatment for HCV was revolutionized when highly effective direct-acting antiviral HCV medications became available in late 2013.^4^ Although these medications cure the majority of patients after only 1 course of treatment, many payers in the United States have found the cost of these medications to be prohibitive, with list prices ranging from $25 000 to $95 000 per prescription.^3,4^

State Medicaid programs, which cover a disproportionate share of adults with HCV,^5,6^ have struggled to balance access to these lifesaving medications with their budget constraints. Owing to the high cost of HCV medications and finite budgets, state Medicaid programs have historically restricted access to HCV treatment.^7^ Nearly all state Medicaid programs limit access to direct-acting antiviral HCV medications through prior authorization requirements and narrow preferred drug lists.^8^ In the 5 years after highly effective HCV medications became available, most states also imposed clinical criteria requiring liver damage, which rationed access to the sickest individuals, and sobriety, which prohibited many with substance use disorders from benefitting from HCV treatment.^1,8-10^

Given rising rates of acute HCV infection and a growing focus on eradicating HCV in the next decade,^11^ alternative payment models for financing HCV medications have come to the forefront as one potential method to increase access. Subscription-based payment models (SBPM), wherein states negotiate reduced prices with a single manufacturer in exchange for exclusivity on drug formularies, may offer a solution that reduces total spending while increasing access to these life-saving medications.^12^ In July 2019, Louisiana and Washington adopted modified SBPMs that use a 2-part pricing strategy. First, both states pay a reduced price per prescription through supplemental rebates up to a certain spending threshold.^13,14^ Second, after hitting this threshold, the per-prescription price falls to near zero through additional supplemental rebates.^13,14^ Under this model, the spending cap provides budget predictability for states and guaranteed revenue for drug manufacturers. This model is attractive for manufacturers of HCV medications, who are facing an increasingly competitive environment as more HCV medications come to market.^15^ In contrast to fee-for-service models, SBPMs remove financial incentives to restrict access to HCV treatment for all those who may benefit, because the marginal cost of additional prescriptions above the threshold is essentially zero (see eFigure 1 in the Supplement).

Subscription-based payment models thus have the potential to constrain per capita spending on high-cost medications while improving population health, an elusive dyad of the Triple Aim.^16^ Proposed by Berwick et al in 2008, the Triple Aim acts as a road map for reforming the US health care system by improving population health, improving the patient experience of care, and reducing per capita cost.^16^ To our knowledge, no research has assessed the effect of these arrangements on use of Medicaid-covered HCV medications. In this cross-sectional study, we used synthetic control models (SCMs) to estimate the association between SBPM implementation and changes in Medicaid-covered HCV prescription fills from 2017 to 2020 in Louisiana and Washington.

## Methods

Our study period ranged from January 1, 2017, to June 30, 2020. Louisiana and Washington both implemented SBPMs on July 1, 2019^13,14^; thus, our preperiod was from January 1, 2017, until June 30, 2019, and our postperiod was from July 1, 2019, until June 30, 2020. Our primary data source for this cross-sectional study was the Centers for Medicare & Medicaid Services’ (CMS) Medicaid State Drug Utilization Data.^17^ States must report utilization of Medicaid-covered prescriptions to CMS as a condition of their participation in the Medicaid Drug Rebate Program. Direct-acting antiviral HCV medications were identified using National Drug Codes according to methods used in previous research (see eTable 1 in the Supplement for full list).^18,19^ We additionally identified authorized generic versions of Epclusa and Harvoni, which were released in 2019.^20^ We included all direct-acting antiviral HCV medications, instead of just those medications included in SBPMs in Louisiana and Washington, to estimate overall changes in prescription fills. By design, SBPMs are expected to dramatically increase utilization of specific HCV medications. Thus, only assessing changes in use of the medications included in SBPM contracts may not reflect changes in total prescription volume.

Adoption of SBPMs was identified using CMS waiver filings, and effective dates were confirmed using state reports (see eTable 2 in the Supplement for documentation). State documents were used to assess the design and structure of SBPMs in Louisiana and Washington.^13,14^ We also obtained data on state characteristics that might plausibly influence use of HCV medications. We identified restrictions imposed by state Medicaid programs to access HCV medications from Hepatitis C: State of Medicaid Access, a collaboration between the Center for Health Law and Policy Innovation at Harvard Law School and the National Viral Hepatitis Roundtable.^9^ Quarterly Medicaid enrollment was obtained from the CMS Monthly Enrollment Reports.^21^ Data on the incidence of acute and chronic HCV infections were obtained from the Centers for Disease Control and Prevention’s National Notifiable Diseases Surveillance System.^22^

This study was determined to not be human participants research by the Boston University Medical Center Institutional Review Board and thus exempt from informed consent. We adhered to the Strengthening the Reporting of Observational Studies in Epidemiology (STROBE) reporting guideline for cross-sectional studies.

### Primary Exposure and Outcome

Our primary exposure was implementation of SBPMs for direct-acting antiviral HCV medications in Louisiana and Washington on July 1, 2019. Our primary outcome was use of Medicaid-covered direct-acting antiviral HCV prescriptions, expressed as a rate of prescription fills per 100 000 Medicaid enrollees per state-quarter.

### Covariates

Liver damage and sobriety restrictions for HCV medication access are frequently imposed by state Medicaid programs. We included these restrictions as covariates given their correlation with use of HCV medications.^23,24^ We considered states requiring any level of liver damage to access HCV medications as having a liver damage requirement. Similarly, we considered states requiring any period of abstinence as having a sobriety requirement. These requirements were coded as binary variables taking on a value of 1 if a state had any restrictions in place during a quarter, or 0 otherwise.^9^

### Statistical Analysis

We used SCMs to estimate the association between SBPM implementation and the fills of Medicaid-covered HCV medications. Synthetic control models are particularly useful when estimating the outcome of a policy change that affects a small number of treatment groups.^25-28^ These methods are similar to traditional difference-in-difference estimation but require fewer assumptions to obtain estimates of association.^28,29^ Difference-in-difference assumes that any differential changes in outcomes between treated and control groups are attributable to the policy change, yet treated and control groups are often nonequivalent in terms of pretreatment outcome levels, trends in outcomes, and other important covariates. To mitigate this limitation, researchers attempt to control for observed variables that may be associated with both treatment likelihood and the outcome of interest. However, treatment and control groups may still differ in terms of outcome pretrends and levels because of unobserved factors. This possibility introduces potential selection issues, which may bias any estimates of association.

In contrast, synthetic control methods constructs a synthetic control from a donor pool of groups not exposed to the treatment of interest.^28^ The synthetic control is constructed using a weighted mean of the control groups, with weights chosen through a data-driven process. Weights for individual control units may range from 0 to 1 and are selected so the synthetic control is as similar as possible to the treated group in terms of outcome pretrends. Unlike traditional regression, inclusion of covariates is not required to achieve equivalence between treated and controls groups. Researchers using synthetic control methods may choose to restrict the donor pool to control groups with similar characteristics or judiciously include covariates deemed most important in the weighting algorithm. However, forcing the SCM algorithm to match across many covariates may worsen the preperiod match on outcome pretrends and levels and is thus not recommended.^28^

Our analysis proceeded in 4 steps. We first created individual SCMs for each treated state (ie, synthetic Louisiana and Washington) from a donor pool of control states.^29^ We limited the donor pool to control states that imposed similar liver damage and sobriety criteria in the quarter prior to SBPM implementation and had majority complete prescription data (<50% suppressed data). We excluded states from the donor pool that did not report coverage for HCV medication access (Illinois) and those that changed the coverage mechanism for Medicaid-covered prescriptions (New Hampshire) during our study period. We also excluded 7 states that had more than 50% suppressed prescription data during the study period (Alaska, Delaware, Iowa, Nebraska, North Dakota, South Dakota, and Wyoming). Three states were missing prescription data for only a single quarter (Arkansas, Montana, and Utah), and these data were replaced through linear interpolation. For each state, weighted means of control states were used to create a synthetic control for each treated state from the donor pool. Weights were selected using a data-driven approach to minimize preperiod differences between treated states and synthetic controls (see eTable 3 and the eMethods in the Supplement for donor pool and synthetic control weights). Our unit of analysis was the state-quarter. Our donor pool included 30 states and the District of Columbia (660 state-quarters).

We then estimated mean differences in Medicaid-covered HCV prescription fills per 100 000 Medicaid enrollees between treated states and synthetic controls in the year following SBPM implementation. Given that SCMs do not produce traditional measures of uncertainty, we employed Taylor series linearization to determine 95% CIs (note that unlike traditional regression analyses, CIs derived in this manner are typically not symmetric around the point estimate).^25,28,30^ We also performed a series of permutation placebo tests to generate placebo effect sizes. For the permutation tests, we reassigned the treatment to each control state and reestimated our SCMs. We then used a 2-sided *t* test to determine whether the observed effect in treated states exceeded the placebo effects in control states at the α = .05 significance level. If so, this provides further evidence that results were unlikely to have occurred by chance. Analyses were conducted using R version 4.0.2 (R Project for Statistical Computing), with SCMs estimated using the microsynth package, version 2.0.17.^31^ More details about these procedures are available in the eMethods in the Supplement.

### Robustness Tests

Several tests were performed to assess the robustness of our results. First, HCV prescription fills were alternatively measured at the per-pill (vs per-prescription) level to account for potential differences in the length of treatment between direct-acting antiviral medications. Second, to account for the emergence of COVID-19 and subsequent disruption in the delivery of health services,^32^ the second quarter of 2020 was excluded from the postperiod. Third, we estimated unrestricted versions of our SCMs, allowing all states to be included in the donor pool instead of only those with similar liver damage and sobriety restrictions. Fourth, we estimated leave-one-out versions of our SCMs to ensure our results were not driven by trends in individual control states. This estimation was achieved by iteratively removing control states from the donor pool and reestimating SCMs.

Louisiana removed restrictions for HCV medication access concomitantly with SBPM implementation. To account for this, we estimated a stacked synthetic control for Louisiana to assess to what extent our results were driven by the removal of liver damage and sobriety restrictions. To construct the stacked SCM, we first limited the donor pool to the 9 states that also removed access restrictions during our study period. Next, we centered the time variable in our data set so that each state’s removal of access restrictions coincided with Louisiana’s SBPM implementation. We then reestimated the SCM, which allowed us to identify the association between SBPM implementation and HCV prescription fills while accounting for changes in access restrictions (see more details on this procedure in the eMethods in the Supplement).

## Results

### SBPMs in Louisiana and Washington

While the exact structural and financial details of SBPMs in each state are confidential, both states use a similar 2-part pricing model as described in the introduction.^13,14^ Louisiana entered into an SBPM with Gilead Sciences Inc subsidiary Asegua Therapeutics for access to the authorized generic of Epclusa for 5 years,^33^ and Washington contracted with AbbVie Inc for access to Mavyret for 5 years.^14^ Louisiana’s SBPM focused on screening and treatment engagement among Medicaid enrollees and incarcerated persons, who in years prior had limited access to HCV medications because of restrictions requiring liver damage and sobriety.^33^ Washington’s SBPM also broadly focused on Medicaid enrollees, but screening and treatment efforts were particularly focused on engaging those who inject drugs (personal communication with officials from the Washington State Health Care Authority [Sullivan D, Fliss M, Evaskus L]; March 10, 2020).^34^ Louisiana and Washington developed similar, robust implementation initiatives designed to scale up screening and treatment capacity.^33,34^ Louisiana aimed to treat and cure 10 000 individuals within the first year of SBPM implementation,^33^ and Washington aimed to treat and cure 4900 individuals within the same time frame.^34^

### Louisiana

Louisiana had liver damage and sobriety restrictions in effect during the preperiod but removed these restrictions concomitantly with SBPM implementation.^13^ Rates of acute HCV infection were stable during the study period, whereas rates of chronic HCV increased from 71.3 to 142.5 cases per 100 000 residents from 2017 to 2018 (eFigure 2 in the Supplement). The rate of chronic HCV decreased to 82.7 cases per 100 000 in 2019. In the year before SBPM implementation in Louisiana, the mean (SD) rate of quarterly HCV prescription fills was 43.1 (8.6) prescriptions per 100 000 Medicaid enrollees. Following SBPM implementation, quarterly HCV prescription fills increased by 162.8 (95% CI, 82.6-243.1), to a mean (SD) of 206.0 (51.2) prescription fills per 100 000 enrollees in Louisiana (Table).

**Figure 1.**
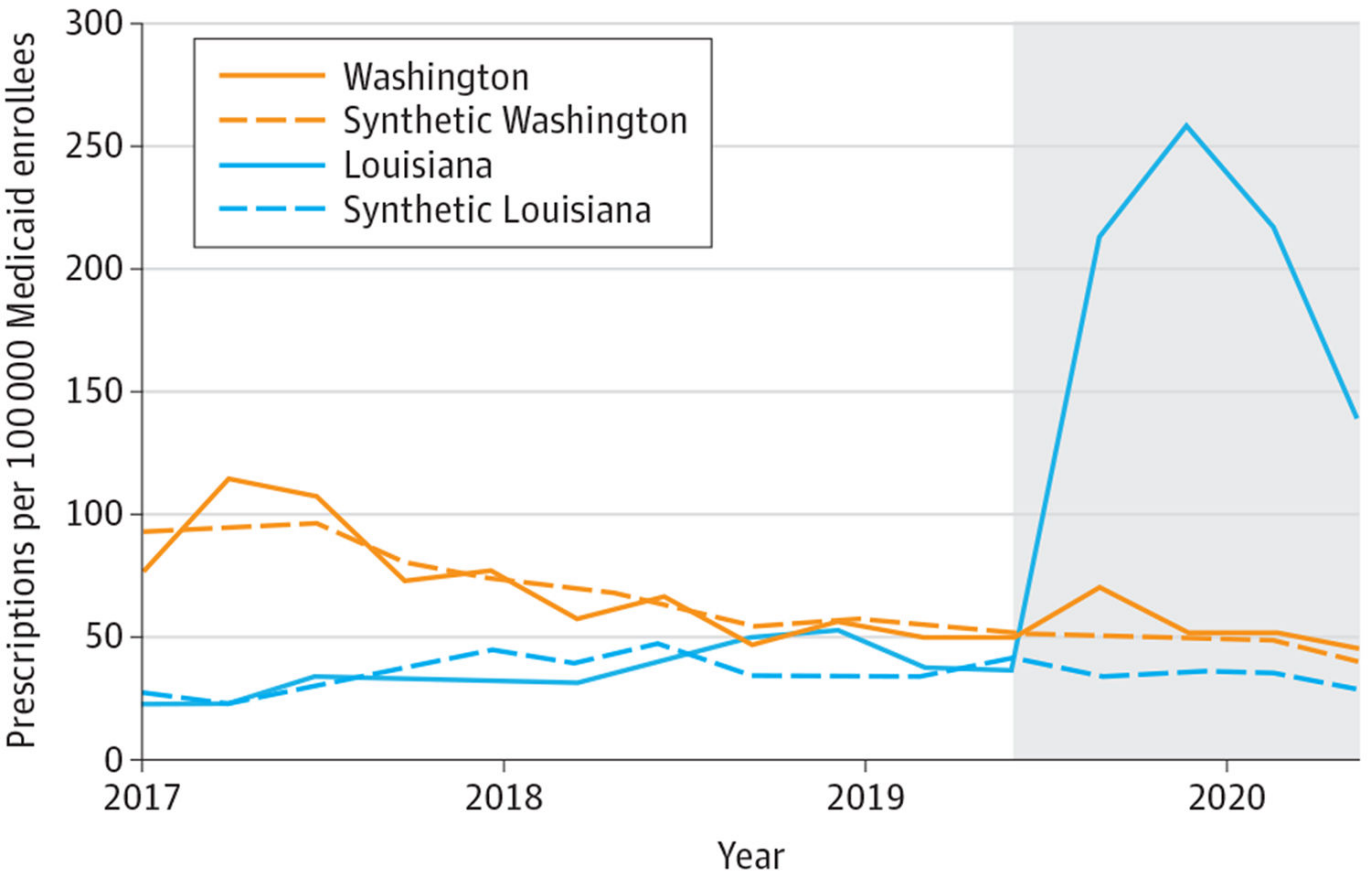
Trends in Hepatitis C Virus Prescription Fills in Treated States and Synthetic Controls This analysis is based on Medicaid State Drug Utilization Data for 2017 through 2020. The shaded section of the graph indicates the post–subscription-based payment model implementation period. Synthetic Louisiana and synthetic Washington are weighted combinations of control states that best approximated pretrends in outcomes and that had similar liver damage and sobriety restrictions. See the eMethods and eTable 3 in the Supplement for a description of the synthetic analysis.

**Figure 2.**
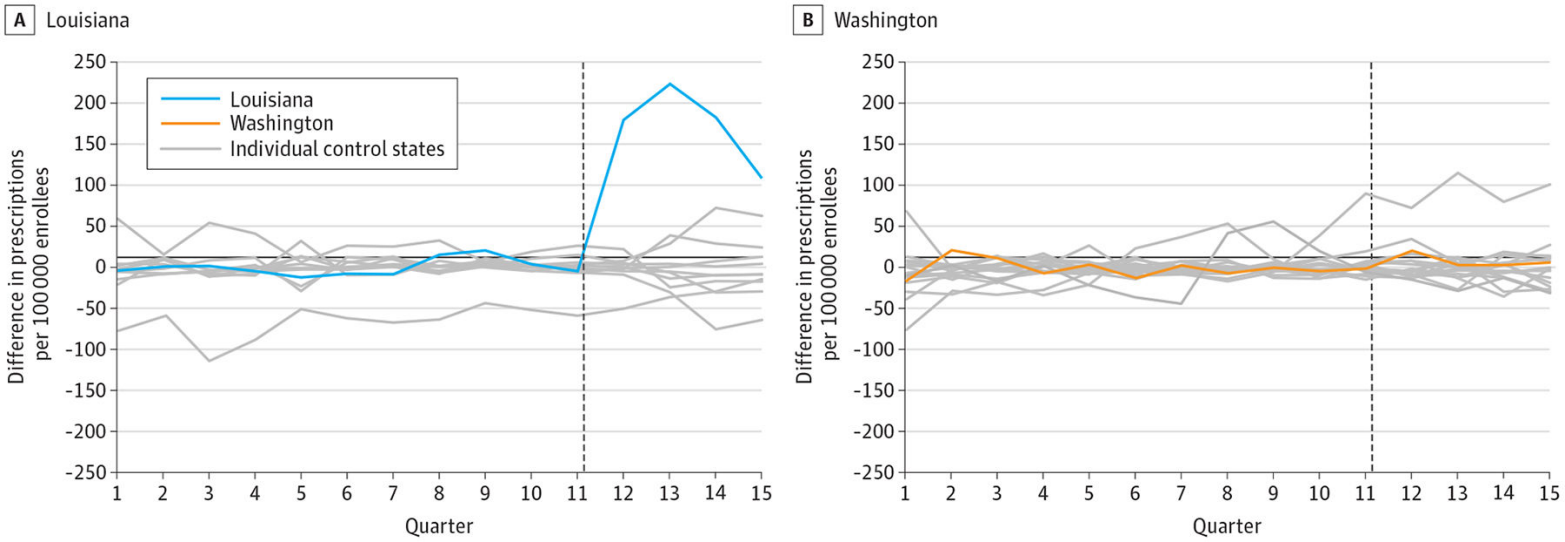
Permutation Tests of Hepatitis C Virus Prescription Fills in Treated States and Synthetic Controls This analysis is based on Medicaid State Drug Utilization Data for 2017 through 2020. The vertical dotted line indicates the start of the post–subscription-based payment model (SBPM) implementation period. The blue and orange lines represent unadjusted trends in utilization for Louisiana and Washington, respectively. Gray lines represent the estimated placebo differences in outcomes between individual control states and their respective synthetic controls. The donor pool of control states was limited to those with similar liver and sobriety restrictions in the quarter immediately preceding SBPM implementation. See the eMethods and eTable 3 in the Supplement for a description of the synthetic analysis.

Pretrends were very similar between Louisiana and its synthetic control (Figure 1). In the preperiod, the mean (SD) absolute difference between Louisiana and its synthetic control was 8.0 (6.4) HCV prescription fills per quarter. Following SBPM implementation, quarterly HCV prescription fills increased by 173.5 (95% CI, 74.3-265.3) per 100 000 Medicaid enrollees compared with its synthetic control, a relative increase of 534.5%(95% CI, 228.7%-1125.0%). Permutation tests indicate our findings in Louisiana were highly unlikely to be due to chance, as test results showed that no other states in the donor pool (N = 11) experienced a greater increase in HCV prescription fills than Louisiana (*P* < .001) (Figure 2).

### Washington

Washington had neither liver damage nor sobriety restrictions in effect to access Medicaid-covered HCV medications during the study period.^9^ Rates of acute and chronic HCV infection were relatively stable during the study period and did not substantially change after SBPM implementation (eFigure 2 in the Supplement). The mean (SD) rate of quarterly HCV prescription fills was 50.1 (4.1) per 100 000 Medicaid enrollees in the year before SBPM implementation and 53.9 (11.0) per 100 000 enrollees in the year after implementation, which was not a significant change (Table).

Very similar pretrends were observed between Washington and its synthetic control. During the preperiod, the mean (SD) absolute difference between Washington and its synthetic control was 7.3 (6.2) HCV prescription fills per quarter. We did not observe a significant change in HCV prescription fills following SBPM implementation (+16.2%; 95% CI, −21.7% to +72.4%).

### Robustness Tests

Our findings were robust to several changes in specification. Results were highly similar when alternatively measuring HCV prescription fills at the per-pill level (vs per prescription). This finding suggests the observed increase in HCV prescription fills was not attributable to differences in treatment length. Our conclusions were also qualitatively unchanged after excluding the second quarter of 2020 from the postperiod to account for the emergence of COVID-19. When including all states in our synthetic control estimation (ie, unrestricted SCM), Louisiana experienced a 702%(95% CI, 362.0%-1292.7%) relative increase in quarterly HCV prescription fills in comparison with its synthetic control (*P* < .001) (eTable 4 in the Supplement). In the leave-one-out analysis, estimates for HCV prescription fills ranged from 515.2% (*P* < .001) to 735.3%(*P* < .001) in Louisiana in comparison with its synthetic control. Finally, results from the stacked model indicate that Louisiana’s quarterly HCV prescription fills increased by 130.2 (95% CI, 82.0-195.4) per 100 000 Medicaid enrollees compared with its synthetic control, a relative increase of 180.2%(95% CI, 114.6%-265.7%).

## Discussion

It has been over a decade since Berwick et al unveiled the Triple Aim, and the US health care system has struggled to improve population health while reducing per capita health care costs.^16^ In this cross-sectional study, SBPMs were associated with a large increase in Medicaid-covered HCV prescription fills in Louisiana but were not associated with changes in use of these medications in Washington. These results suggest that SBPMs have the potential to improve population health by increasing access to HCV medications while capping spending—a critical but elusive dyad of the Triple Aim.^16^

The heterogenous response of these 2 states to implementation of a SBPM may be partially attributable to historical access to direct-acting antiviral HCV medications, differences in SBPM implementation, and the onset of the COVID-19 pandemic. Washington removed liver damage and sobriety restrictions in late 2016^9^ and did not experience a notable increase in use with its SBPM. In contrast, Louisiana experienced a substantial increase in use of HCV medications after SBPM implementation but concurrently removed these restrictions. However, in a synthetic control model constructed only from states that also removed these restrictions, Louisiana experienced a greater than 180% relative increase in prescription fills. Thus, removing liver damage and other access restrictions may be necessary but not sufficient to dramatically increase use of HCV medications. It is also plausible that the spending cap of the SBPM enabled the change in access criteria in Louisiana. The spending cap removes states’ incentives to ration access to HCV medications, which may encourage the adoption of policies that facilitate rather than inhibit access to HCV treatment. Nonetheless, the disparate influence of SBPMs in Louisiana and Washington suggests that states with greater pent-up demand for HCV medications may expect to see larger gains in use from a SBPM.

Subscription-based payment model implementation initiatives may have also driven differential state-level changes in use. Although both Louisiana and Washington included plans to increase screening and treatment for HCV, the effectiveness of these initiatives are unknown. It is possible that Louisiana was better able to scale up screening and treatment initiation for HCV, which may have driven the observed changes in use. Notably, reported chronic HCV cases increased 99% from 2017 to 2018 in Louisiana, then declined 42% from 2018 to 2019. The uptick in reported cases of chronic HCV may reflect an effort to screen more individuals for HCV in Louisiana in preparation for SBPM implementation. Moreover, given the access restrictions in place prior to the SBPM, many individuals in Louisiana may have been aware they were infected with HCV but did not previously qualify for treatment. Washington intended to focus on traditionally hard-to-reach populations and planned to offer HCV testing at needle exchanges and substance use disorder treatment facilities, but these initiatives were halted by the onset of the COVID-19 pandemic (personal communication with officials from the Washington State Health Care Authority [Sullivan D, Fliss M, Evaskus L]; March 10, 2020). Other initiatives, such as promotional testing buses that were going to be driven around the state, were also halted because of the pandemic (personal communication with officials from the Washington State Health Care Authority [Sullivan D, Fliss M, Evaskus L]; March 10, 2020). Stalled implementation efforts may have hindered Washington’s ability to identify those with HCV and engage them in treatment. Moreover, Washington experienced a larger decline in mobility than Louisiana in the first months of the pandemic, which may reflect differences in state policies aimed to reduce spread.^35^ As such, differences in state-level responses to COVID-19 may have also unintentionally hindered HCV screening, disease identification, and medication access.

### Limitations

Our study has several limitations. First, we used only publicly available data sources and did not have access to individual-level data. Thus, we were unable to account for the prevalence of HCV among Medicaid enrollees or the frequency of HCV testing in Louisiana and Washington. Our primary data source, the Medicaid State Drug Utilization Data, suppresses records with fewer than 11 prescriptions per quarter, predominantly affecting rural states with small populations. Thus, our results may not generalize to rural states. Second, our study design allowed us to estimate the overall association between SBPMs and changes in prescription fills for Washington and Louisiana separately. Therefore, we cannot make direct comparisons between them given that each state has a unique synthetic control. Third, we were unable to speak to granular differences in the implementation or contractual details of SBPMs in either state because this information is not publicly available. Fourth, the observational nature of our study limits causal conclusions regarding the effects of SBPMs on use of Medicaid-covered HCV medications. Finally, we cannot speak to the effect of SBPMs on spending related to HCV treatment.

## Conclusions

In this cross-sectional study, SBPMs were associated with increased Medicaid-covered HCV prescription fills in Louisiana but not in Washington. These results suggest that SBPMs may enable states to facilitate access to effective but costly medications, which may both improve the health of HCV-infected Medicaid enrollees and potentially reduce downstream spending on complications from untreated HCV.

## Supplementary Material

Supplemental Materials **eFigure 1.** Conceptual model of drug spending under fee-for-service vs subscription-based payment models
**eTable 1.** National Drug Codes used to identify direct-acting antiviral HCV medications
**eTable 2.** Policy effective dates for implementation of SBPMs in Louisiana and Washington
**eMethods.** Additional Methodology
**eTable 3.** States with Non-Zero Weight in Synthetic Controls
**eFigure 2.** Trends in rates of acute and chronic HCV infections in treated states and nationally
**eTable 4.** Unrestricted Changes in Medicaid-covered HCV prescription fills associated with implementation of subscription-based payment models

## Figures and Tables

**Table.** Changes in Medicaid-Covered Hepatitis C Virus Prescription Fills per 100 000 Medicaid Enrollees Associated With Implementation of Subscription-Based Payment Models (SBPMs)

	Unadjusted quarterly prescription fills, mean (SD)^a^		Synthetic control estimates^b^	
Outcome	Pre-SBPM	Post-SBPM	Change (95% CI), %	Linear *P* value
Louisiana	43.1 (8.6)	206.0 (51.2)	534.5 (228.7 to 1125.0)	<.001
Washington	50.1 (4.1)	53.9 (11.0)	16.2 (−21.7 to 72.4)	.42

a Mean quarterly Medicaid-covered hepatitis C virus prescription fills during the 4 quarters immediately preceding and following SBPM adoption.

b Synthetic control estimates for the percent change in prescription fills during the 4 quarters after adoption. Refer to the eMethods and eTable 3 in the Supplement for a description of synthetic analysis.

## Key Points

### Question

Did the use of direct-acting antiviral hepatitis C virus (HCV) medications change after implementation of subscription-based payment models for these drugs in Washington and Louisiana?

### Findings

In this cross-sectional study, Louisiana experienced a 534.5% increase in HCV prescription fills after implementation of a subscription-based payment model, but no significant change in prescription fills was observed in Washington.

### Meaning

In this study, subscription-based payment models in Louisiana and Washington were differentially associated with use of Medicaid-covered HCV medications, which may reflect state-level differences in implementation, historical restrictions on access to these medications, and responses to the COVID-19 pandemic.
