# Supplementary material for: Medicaid Subscription-Based Payment Models and Implications for Access to Hepatitis C Medications
Source: JAMA Health Forum. Author manuscript; Available in PMC 2022 Feb 7. (PMC8796990; doi:10.1001/jamahealthforum.2021.2291)
Supplement: Supplemental Materials — eFigure 1. Conceptual model of drug spending under fee-for-service vs subscription-based payment models eTable 1. National Drug Codes used to identify direct-acting antiviral HCV medications eTable 2. Policy effective dates for implementation of SBPMs in Louisiana and Washington eMethods. Additional Methodology eTable 3. States with Non-Zero Weight in Synthetic Controls eFigure 2. Trends in rates of acute and chronic HCV infections in treated states and nationally eTable 4. Unrestricted Changes in Medicaid-covered HCV prescription fills associated with implementation of subscription-based payment models [file NIHMS1761375-supplement-Supplemental_Materials.pdf]

Supplemental Online Content

Auty SG, Shafer PR, Griffith KN. Medicaid subscription-based payment models and implications for access to hepatitis C medications. *JAMA Health Forum*. 2021;2(8):e212291. doi:10.1001/jamahealthforum.2021.2291

eFigure 1. Conceptual model of drug spending under fee-for-service vs subscription-based payment models

eTable 1. National Drug Codes used to identify direct-acting antiviral HCV medications

eTable 2. Policy effective dates for implementation of SBPMs in Louisiana and Washington

eMethods. Additional Methodology

eTable 3. States with Non-Zero Weight in Synthetic Controls

eReferences.

eFigure 2. Trends in rates of acute and chronic HCV infections in treated states and nationally.

eTable 4. Unrestricted Changes in Medicaid-covered HCV prescription fills associated with implementation of subscription-based payment models

This supplemental material has been provided by the authors to give readers additional information about their work.

© 2021 Auty SG et al. *JAMA Health Forum.*

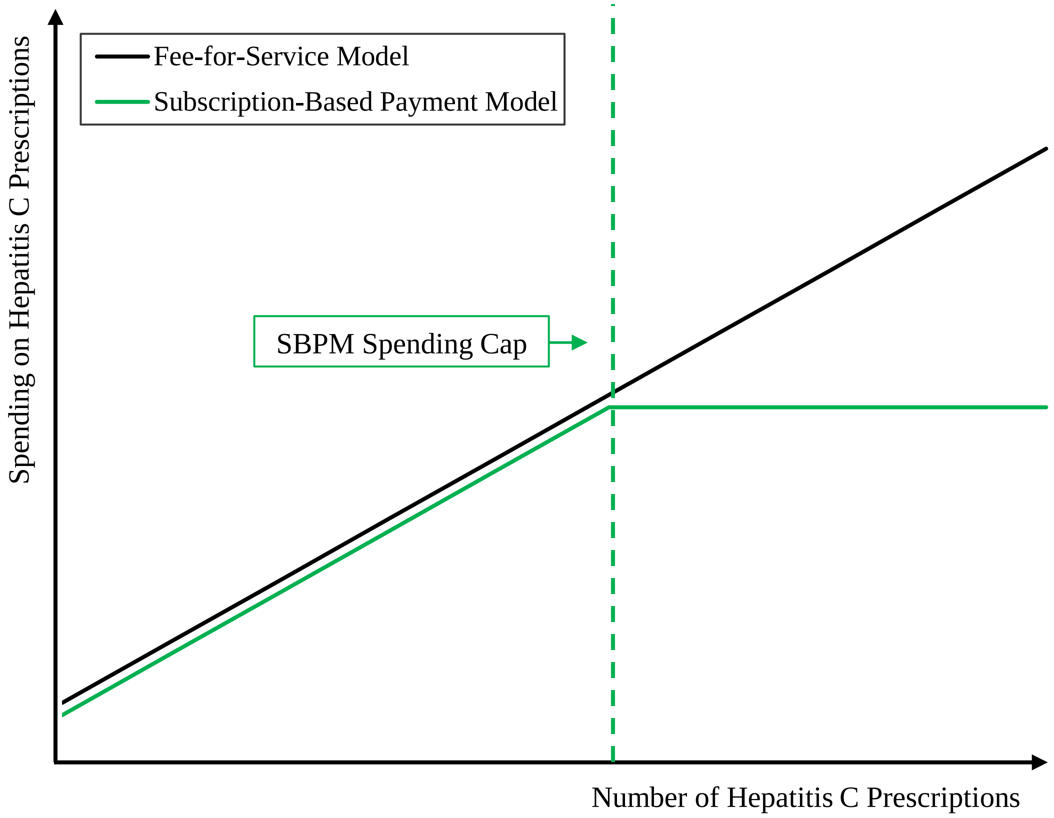

eFigure 1. Conceptual model of drug spending under fee-for-service vs subscription-based payment models

Source/Notes: Author's visualization.

© 2021 Auty SG et al. *JAMA Health Forum.*

eTable 1. National Drug Codes used to identify direct-acting antiviral HCV medications

**Drug Name**	**Active Ingredients**	**11-Digit NDC**
Sovaldi	Sofosbuvir	61958150101
Sovaldi	Sofosbuvir	61958150201
Sovaldi	Sofosbuvir	61958150202
Sovaldi	Sofosbuvir	61958150301
Sovaldi	Sofosbuvir	61958150401
Sovaldi	Sofosbuvir	61958150501
Harvoni	Ledipasvir/sofosbuvir	61958180501
Harvoni	Ledipasvir/sofosbuvir	61958180401
Harvoni	Ledipasvir/sofosbuvir	61958180301
Harvoni	Ledipasvir/sofosbuvir	61958180202
Harvoni	Ledipasvir/sofosbuvir	61958180101
Viekira Pak	Ombitasvir/paritaprevir/ritonavir/dasabuvir	00074309328
Viekira Pak	Ombitasvir/paritaprevir/ritonavir/dasabuvir	00074309301
Technivie	Ombitasvir/paritaprevir/ritonavir	00074308228
Zepatier	Elbasvir/grazoprevir	00006307402
Zepatier	Elbasvir/grazoprevir	00006307401
Epclusa	Sofosbuvir/velpatasvir	61958220101
Epclusa	Sofosbuvir/velpatasvir	61958220201
Epclusa	Sofosbuvir/velpatasvir	61958220202
Epclusa	Sofosbuvir/velpatasvir	61958220203
Epclusa	Sofosbuvir/velpatasvir	61958220301
Viekira XR	Dasabuvir/ombitasvir/paritaprevir/ritonavir	00074006328
Viekira XR	Dasabuvir/ombitasvir/paritaprevir/ritonavir	00074006301
Vosevi	Sofosbuvir/velpatasvir/voxilaprevir	61958240101
Mavyret	Glecaprevir/pibrentasvir	00074262528
Mavyret	Glecaprevir/pibrentasvir	00074262501
Mavyret	Glecaprevir/pibrentasvir	00074262556
Mavyret	Glecaprevir/pibrentasvir	00074262580
Epclusa authorized generic	Velpatasvir/sofosbuvir	72626270101
Harvoni authorized generic	Ledipasvir/sofosbuvir	72626260101

© 2021 Auty SG et al. *JAMA Health Forum.*

eTable 2. Policy effective dates for implementation of SBPMs in Louisiana and Washington

State	Effective Date	URL
Louisiana	7/1/2019	https://www.medicaid.gov/sites/default/files/State-resource-center/Medicaid-State-Plan-Amendments/Downloads/LA/LA-19-0018.pdf
Washington	7/1/2019	https://www.doh.wa.gov/Portals/1/Documents/Pubs/150nonDOH-HepCFreeWA-PlanJuly2019.pdf

eMethods. Additional Methodology

Sample and Measurement of Outcome Variables

The pre-period for our analysis ran from January 1, 2017 to June 30th, 2019, and our post-period ran from July 1, 2019 through June 30th, 2020. We required control states to have similar liver damage and sobriety restrictions to access HCV medications as treated states in the quarter immediately preceding SBPM implementation. Thus, the "donor pool" for Louisiana included 11 control units that had both liver and sobriety restrictions in the second quarter of 2019 (Alabama, Arkansas, District of Columbia, Georgia, Louisiana, Michigan, Minnesota, Oklahoma, Texas, West Virginia, Wisconsin). The donor pool for Washington included 19 control units that had neither liver nor sobriety restrictions in the second quarter of 2019 (California, Colorado, Connecticut, Idaho, Maine, Massachusetts, Missouri, Nevada, New Mexico, New York, North Carolina, Ohio, Oregon, Pennsylvania, Rhode Island, South Carolina, Vermont, Virginia, Washington). Our final analytic dataset included 660 quarters of data from 30 states and the District of Columbia. The Table shows the weights of states with non-zero weights in each synthetic control model.

eTable 3. States with Non-Zero Weight in Synthetic Controls

	**Analytic Weight**	
**Control state**	**Synthetic Louisiana**	**Synthetic Washington**
Alabama	--	10.4
Connecticut	12.9	--
District of Columbia	--	2.9
Michigan	--	3.7
Nevada	25.5	--
New Mexico	12.0	--
Oklahoma	--	5.1
South Carolina	49.6	--
Texas	--	40.5
Wisconsin	--	37.4

We calculated our outcome variables quarterly as follows:

$$\text{HCV prescription fills per 100,000 enrollees } = \frac{Total\ HCV\ prescriptions}{Total\ Medicaid\ enrollees} \times 100{,}000$$

Unadjusted Comparisons

Unadjusted changes in HCV prescription fills were identified by comparing the four calendar quarters preceding implementation of subscription-based payment-models (July 1st, 2018 through June 30th, 2019) with the following four quarters (July 1st, 2019 through June 30th, 2020). Significance levels and confidence intervals were calculated using two-sided t-tests.

Synthetic Control Comparisons

The basic idea of synthetic control methods is to use a weighted average of each state's donor pool, with the weights chosen so that pre-trends in outcomes are as similar as possible between the treated state(s) and synthetic control.[1] We followed the approach outlined in Robbins, Saunders, and Kilmer (2016) and implemented in package 'microsynth' for R Statistical Software version 4.0.2.[2] We estimated separate synthetic controls models for each treated state and study outcome, for a total of four models.

© 2021 Auty SG et al. *JAMA Health Forum.*

Taylor series linearization (TSL) was then used to calculate estimates and 95% confidence intervals for the effects of SBPM implementation during the following year. This approach accounts for the complex weighting of control states used to create the synthetic control. TSL uses a linear function of the observed data to approximate the estimator, and the variance estimation formulae for a linear estimator can then be applied to the linear approximation. In general, this leads to a statistical consistent estimator of the variance.[3] The TSL method tells us whether any differences in outcome between the treated states and their synthetic controls are statistically significant.

We also conducted a series of permutation tests to determine placebo effect sizes. This procedure occurs in four steps. First, we subset the donor pool of control states for a given treated state and outcome. Second, we iteratively reassign the "treatment" to each control state. Third, weights are calculated to match the placebo treatment to a new synthetic control, and a placebo effect, sampling distribution and associated p-value are generated. Lastly, we use a 2-sided t-test to determine whether the observed effect in the treated state is likely to have occurred by chance, given the distribution of placebo effect estimates.[4]

Robustness Tests

Several tests were performed to assess the robustness of results:

(1) In our main specification, our primary outcome was the rate of HCV prescription fills per 100,000 Medicaid enrollees. We alternatively measured out outcome at the per-pill level to account for potential differences in the length of treatment between direct-acting antiviral medications.

(2) We re-estimated our synthetic control models excluding the second quarter of 2020. This enabled us to assess the sensitivity of our results to the emergence of COVID-19 and subsequent disruption in the delivery of health services.

(3) In our main specification, we curated the donor pools for Louisiana and Washington to include only those states which had similar liver damage and sobriety restrictions in place at the time of SBPM implementation. Alternatively, we estimated 'unrestricted' versions of our synthetic control models allowing all control states to be included in the donor pool.

(4) To test whether our results are driven by trends in individual control states, we estimated 'leave one out' versions of our synthetic control models. This was achieved by iteratively removing control states from the donor pool and re-estimating our synthetic control models.

(5) Louisiana removed restrictions on HCV medication access (i.e., liver damage and sobriety restrictions) concomitantly with SBPM implementation. The SBPM granted the state budget predictability and may have enabled the loosening of access restrictions; nonetheless we estimated a 'stacked' synthetic control model for Louisiana to assess to what extent changes in HCV medication utilization were associated wither SBPM implementation versus loosening of access restrictions.

    The stacking procedure proceeded as follows. We first limited the donor pool to those states that also removed both liver damage and sobriety restrictions during our study period. Next, we manipulated the time variable in our data set, shifting states forward or backward so their loosening of access restrictions coincides with SBPM implementation (graphically depicted in eFigure 3). Additionally, states were removed if they lacked either two years of pre-period data or

© 2021 Auty SG et al. *JAMA Health Forum.*

one year of post-period data after shifting. The final donor pool included Maine, Missouri, Nevada, Ohio, Oregon, Rhode Island, Vermont, & Wisconsin. We then re-estimated our synthetic control model using this manipulated dataset. Since all control states now removed restrictions on HCV medication access concurrently with Lousiana's SBPM implementation, these results allowed us to identify the association between SBPM implementation and HCV prescription utilization while accounting for changes in access restrictions.

eReferences

1. Athey S, Imbens GW. The state of applied econometrics: Causality and policy evaluation. *J Econ Perspect*. 2017;31(2):3-32.

2. Robbins MW, Saunders J, Kilmer B. A framework for synthetic control methods with high-dimensional, micro-level data: evaluating a neighborhood-specific crime intervention. *J Am Stat Assoc*. 2017;112(517):109-126.

3. Lavrakas P. Encyclopedia of Survey Research Methods. 2008. doi:10.4135/9781412963947 NV - 0

4. Robbins MW, Davenport S. microsynth: Synthetic Control Methods for Disaggregated and Micro-Level Data in R. *J Stat Software; Vol 1, Issue 2* . January 2021. https://www.jstatsoft.org/v097/i02.

© 2021 Auty SG et al. *JAMA Health Forum.*

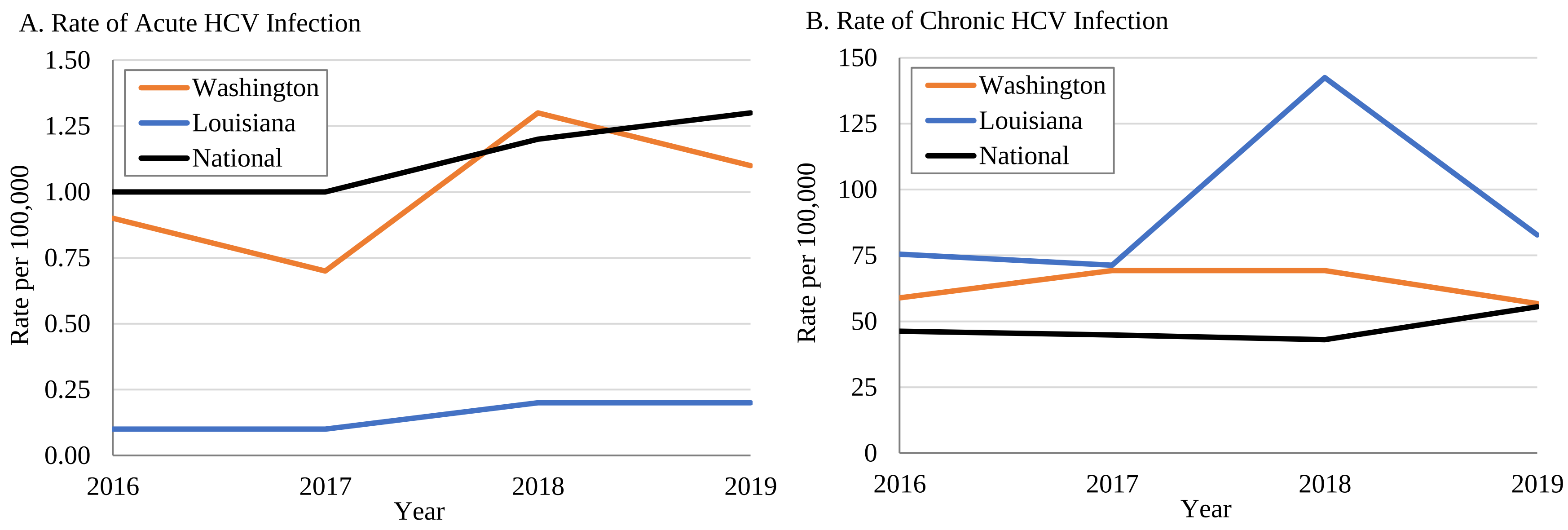

eFigure 2. Trends in rates of acute and chronic HCV infections in treated states and nationally.

© 2021 Auty SG et al. *JAMA Health Forum.*

eTable 4. Unrestricted Changes in Medicaid-covered HCV prescription fills associated with implementation of subscription-based payment models					
**Outcome**	**Unadjusted Quarterly Means[a], (SD)**			**Synthetic Control Estimates[b]**	
	**Pre-SBPM**	**Post-SBPM**		**% Change (95% CI)**	**Linear P-value**
**Louisiana**					
HCV prescriptions[c]	43.1 (8.6)	206.0 (51.2)		702.1% (362.0%, 1292.7%)	<0.001
**Washington**					
HCV prescriptions[c]	50.1 (4.1)	53.9 (11.0)		38.5% (-29.0%, 170.4%)	0.260

[a] Mean quarterly outcome during the 12 months immediately preceding and following SBPM adoption.
[b] Synthetic control estimates for the % change in outcomes during the four quarters after SBPM adoption.
[c] Per 100,000 Medicaid enrollees

© 2021 Auty SG et al. *JAMA Health Forum.*
